# Naringin increases osteoprotegerin expression in fibroblasts from periprosthetic membrane by the Wnt/β-catenin signaling pathway

**DOI:** 10.1186/s13018-020-02145-z

**Published:** 2020-12-10

**Authors:** Chao Yang, Wei Liu, Xianlong Zhang, Bingfang Zeng, Yebin Qian

**Affiliations:** Department of Orthopaedics, Shanghai Jiao Tong University Affiliated Sixth People’s Hospital, 600 Yishan Road, Shanghai, 200233 China

**Keywords:** Naringin, Osteoprotegerin, Wnt/β-catenin, Fibroblasts, Aseptic loosening

## Abstract

**Background:**

The osteoclast bone resorption is critical in aseptic loosening after joint replacement. The balance between activator of nuclear factor kappa B ligand (RANKL) and osteoprotegerin (OPG) is considered to play a central role in osteoclast maturation. Fibroblasts from the periprosthetic membrane express RANKL and promote osteoclast formation. Studies have demonstrated that naringin inhibited osteoclastogenesis and wear particle-induced osteolysis. In this study, the naringin-induced OPG/RANKL effects and its underlying mechanism were studied in fibroblasts from periprosthetic membrane.

**Methods:**

Fibroblasts were isolated from the periprosthetic membrane during hip arthroplasty for revision due to aseptic loosening. Fibroblasts were cultured and treated with or without naringin and DKK-1 (the classical inhibitor of Wnt/β-catenin signaling pathway). OPG and RANKL mRNA and protein levels, gene expression of β-catenin, and cyclin D1, which participate in the Wnt signaling pathway, were examined by real-time polymerase chain reaction and enzyme-linked immunosorbent assay.

**Results:**

The mRNA and protein levels of OPG were enhanced by naringin in a dose-dependent manner compared to that of the non-treated control. In contrast, naringin did not affect the expression of RANKL. Importantly, DKK-1 attenuated OPG expression in fibroblasts under naringin treatment. Moreover, naringin stimulated the gene expression of β-catenin and cyclin D1 in fibroblasts, and the effect could be inhibited by DKK-1.

**Conclusion:**

The results indicated that naringin enhanced OPG expression through Wnt/β-catenin signaling pathway in fibroblasts from periprosthetic membrane, which may be useful to inhibit periprosthetic osteolysis during aseptic loosening after total joint arthroplasty.

## Background

Total hip replacement is one of the most effective treatments for advanced joint diseases. Periprosthetic osteolysis and subsequent aseptic prosthetic loosening are still the most common complications that limit the life of prostheses. The osteolysis is related to a chronic inflammation caused by wear debris gathering between the implant and bone. The inflammation is characterized by a granulomatous membrane at the prosthetic interface that is infiltrated with macrophages, fibroblasts, and giant cells [1].

The RANKL, which is a member of the tumor necrosis factor family, has been shown to support osteoclast differentiation and maturation. It was found that the RANKL expression was enhanced in the tissues around the failed prostheses [2]. The pseudosynovial fluid of loosened total hip prostheses contained high levels of RANKL and could induce osteoclast formation [3].

Although fibroblasts make up most of the cells at the membrane interface, there is less information about the role of fibroblasts in aseptic loosening than other cell types such as macrophages, osteoblasts, and osteoclasts. It has been shown that fibroblasts of periprosthetic membrane are an important source of RANKL [1, 4–8]. The expression of RANKL by fibroblasts in periprosthetic membrane promoted osteoclastogenesis and played a key role in wear debris-induced osteolysis. In the mouse model, loss of RANK-RANKL signal could lead to complete absence of osteolysis caused by wear debris [9]. OPG inhibited the interaction between RANKL and RANK, thus preventing wear debris-induced osteolysis [10, 11].

The Wnt/β-catenin signaling pathway may play an important role in regulating RANKL/OPG expression. Several studies have shown that OPG was increased through Wnt/β-catenin signaling pathway [12–15].

Bisphosphonates are considered to be effective drugs to control osteolytic diseases. We previously reported that alendronate stimulated OPG expression in fibroblasts from periprosthetic membrane [16]. However, bisphosphonates have adverse effects such as gastrointestinal irritation, atypical femur fractures, or even osteonecrosis of the jaw. Therefore, new biological agents are urgently needed for osteolysis caused by wear debris.

Naringin is a natural product, chemically 4′,5,7-trihydroxyflavanone-7-rhamnoglucoside. It is a major flavanone glycoside extracted from tomatoes, grapefruits, and many other citrus fruits. Naringin has diverse biological and pharmacological properties, including anti-inflammatory, anti-oxidant and anti-apoptotic activities. Previous studies have shown that naringin could promote osteoblastogenesis and inhibited osteoclastogenesis [17–25]. Recent studies have shown that naringin significantly increased the expression of OPG in osteoblasts and human amniotic fluid-derived stem cells [20, 24].

Most of studies on naringin have concentrated on its effects on osteoblasts and osteoclasts. However, the effect of naringin on fibroblasts is rarely concerned. In previous studies, we found that fibroblasts in the periprosthetic membrane played a role in aseptic loosening and expressed RANKL and OPG [26–28]. In the present work, the naringin-induced OPG/RANKL effects and its underlying mechanism were studied in fibroblasts from periprosthetic membrane.

## Methods

### Cell culture

The femoral and acetabular pseudomembrane samples were collected from eight patients (5 females and 3 males; mean age 72 ± 10 years), near osteolytic lesions between the implants or cement and bone, who underwent revision due to aseptic loosening of total hip arthroplasty. Every patient presented with a loose prothesis with radiological osteolysis. No patient had clinical or laboratory signs of infection. The indication of primary total hip replacement was osteoarthritis in all patients. Three of the implants were cemented, and five were uncemented. The mean years in situ were 11 ± 4 years. Informed consent was obtained from all donors, and the institutional ethical committee approved the procedures. The samples were washed with PBS, cut into small pieces, and digested in α-minimal essential medium (α-MEM) (Gibco) which contained 1 mg/mL collagenase (Sigma), added with 100 mg/mL of penicillin-G (Sigma), 50 mg/mL of gentamicin sulfate (Sigma), at 37 °C for 30 min. They were then incubated in Versene (Invitrogen) for 1 h [8]. The cells were resuspended in α-MEM containing 10% heat-inactivated fetal bovine serum (Gibco). These cells were cultured at 37 °C in humidified air containing 5% carbon dioxide and then cultured for four generations before the subsequent experiments.

### Phenotype evaluation of cultures

The periprosthetic membrane-derived cells were immunochemically stained with the following antibodies to determine the phenotype evaluation and purity of the culture: fibroblast marker vimentin (Abcam), leukocyte/macrophage marker, CD45 and CD14 (Dako), endothelial cell marker CD31 (Dako). In order to determine the presence of osteoblasts in cultured cells, the histochemical evaluation of alkaline phosphatase expression in the cultures was also carried out.

### Cell proliferation assay

Cell proliferation was analyzed by Cell Counting Kit-8 (CCK-8) method. Fibroblasts (1 × 104/well) were seeded into a 96-well plate and treated at various concentrations (0, 50, 100, or 150 μ M) of narinign for 24 or 48 h. The cells were washed 3 times in PBS and cultured for another 4 h with 10% CCK-8-containing medium. Then, the absorbance at 450 nm was measured using a microtablet reader.

### Stimulation of fibroblasts

Confluent-stage fibroblasts were cultured in fresh medium containing 2% heat-inactivated fetal bovine serum, with or without naringin (Sigma-Aldrich St. Louis, MO, USA) and DKK-1 (Peprotech, USA).

### Real-time quantitative PCR

The fibroblasts were cultured and treated with 0, 50, 100, or 150 μM naringin in the presence or absence of 100 ng/ml DKK-1 for 6 h. Total RNA was isolated with TRIzol reagent (Invitrogen) and converted to cDNA using the SuperScript First-Strand Synthesis Kit (Invitrogen). PCR was performed using the QuantiTect SYBR Green PCR Kit (Toyobo, Osaka, Japan) and the 7500 Real-Time PCR Detection System (Applied Biosystems, USA). OPG was amplified using 5′-CGC CTC CAA GCC CCT GAG GT-3′, 5′-CAA GGG GCG CAC ACG GTC TT-3′; RANKL was amplified using 5′-GTC TGC AGC GTC GCC CTG TT-3′, 5′-ACC ATG AGC CAT CCA CCA TCG C-3′; β-catenin was amplified using 5′-TTG AAA ATC CAG CGT GGA CA-3′, 5′-TCG AGT CAT TGC ATA CTG TC-3′; Cyclin D1 was amplified using 5′-ACA AAC AGA TCA TCC GCA AAC AC-3′, 5′ TGT TGG GGC TCC TCA GGT TC-3′; and β-actin was amplified using 5′-AGG CCA ACC GCG AGA AGA TGA CC-3′, 5′-GAA GTC CAG GGC GAC GTA GCA C -3′ primer sets.

### RANKL and OPG enzyme-linked immunosorbent assay

For RANKL and OPG detection, fibroblasts were treated with naringin (0 μM, 50 μM, 100 μM, 150 μM) in the presence or absence of 100 ng/ml DKK-1 for 24 h. The levels of RANKL and OPG in the culture supernatants were determined by commercially available enzyme-linked immunosorbent assay kits (R&D Systems) in accordance with the manufacturer’s instructions.

### Statistical analysis

Results were representative of at least three independent experiments. The data were expressed as means ± standard deviations (SD). Student’s *t* test was used to determine the statistical significance. The normal distribution was determined by the Kolmogorov-Smirnov test. The data were analyzed using one-way analysis of variance (ANOVA). A *P* value less than 0.05 was considered statistically significant.

## Results

### Phenotype of the cells

After four passages, almost all of the cultures were spindle mononuclear cells. These cells were positive for vimentin and negative for CD45, CD14, CD31, and alkaline phosphatase.

### Cell proliferation

**Fig. 1:**
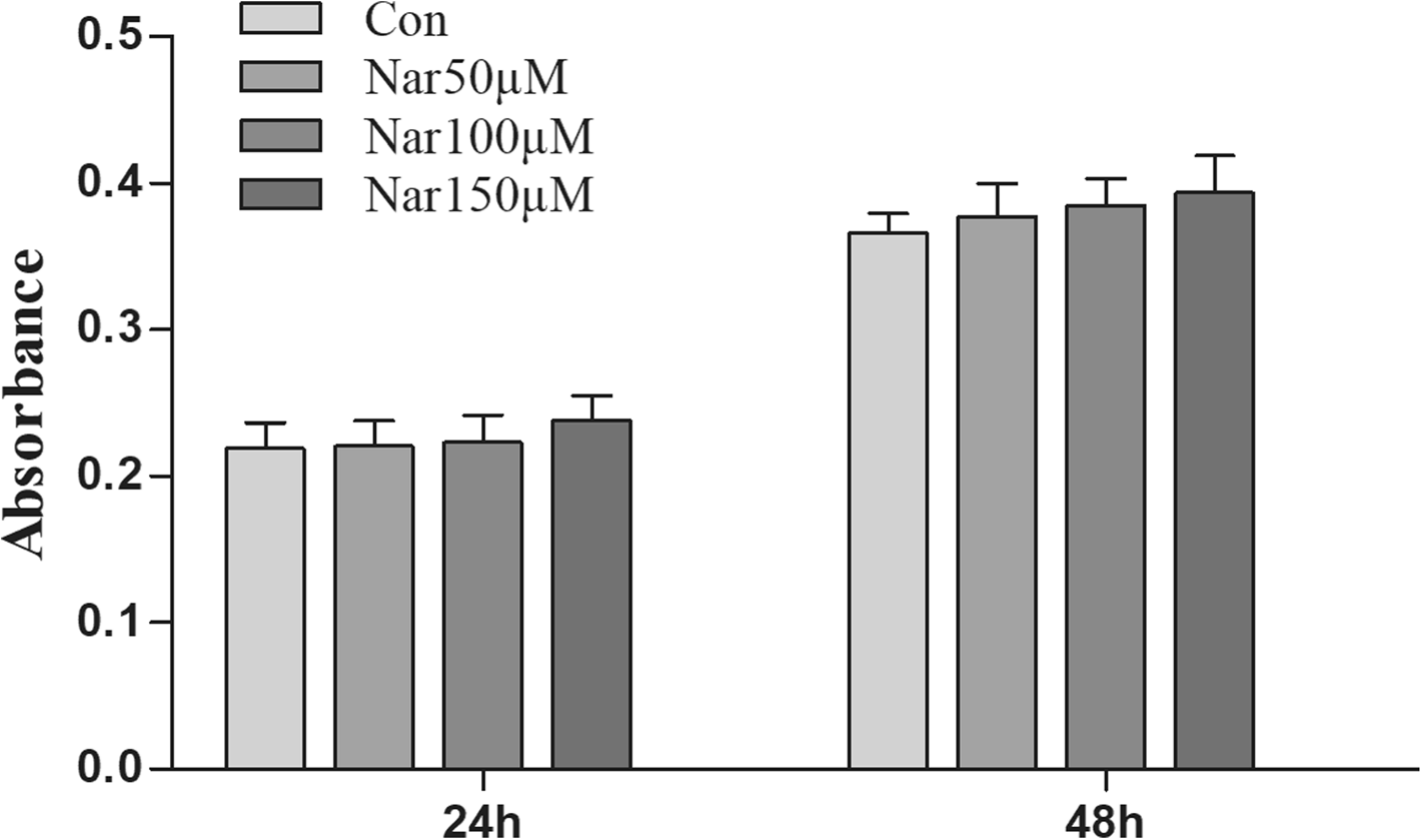
CCK-8 results of fibroblasts cultured on different groups for 24 and 48 h. There was no obvious difference among the four groups (con, 50 μM, 100 μM, 150 μM naringin)

The result of cell proliferation is presented in Fig. 1. After incubation for 24 h and 48 h, no obvious difference was reported among the four groups (con, 50 μM, 100 μM, 150 μM naringin) suggesting that naringin was not obviously cytotoxic to fibroblasts.

### Naringin increased OPG mRNA of fibroblasts from periprosthetic membrane

**Fig. 2:**
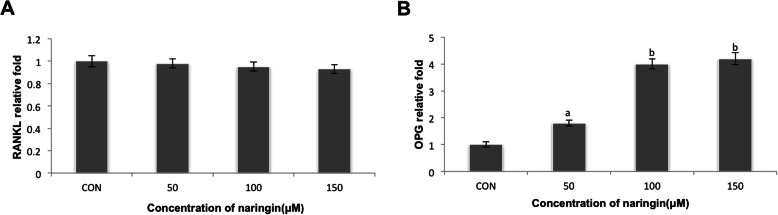
Naringin stimulated OPG mRNA in hip periprosthetic membrane-derived fibroblasts. The fibroblasts were treated with naringin at various concentrations (50 μM, 100 μM, 150 μM) or in medium alone (control group) for 6 h. **a** RANKL mRNA and **b** OPG mRNA expression was examined by real-time RT-PCR. Naringin did not increase RANKL mRNA but enhanced OPG mRNA in a dose-dependent manner (ap < 0.01, bp < 0.001)

The fibroblasts were cultured with naringin (50 μM, 100 μM, 150 μM) or only in the medium (control group) for 6 h. As shown in Fig. 2a and b, real-time RT-PCR showed that the expression of RANKL mRNA was not affected, while the expression of OPG mRNA was enhanced in a dose-dependent manner (ap < 0.01, bp < 0.001).

### Naringin increased OPG secretion of fibroblasts from periprosthetic membrane

**Fig. 3:**
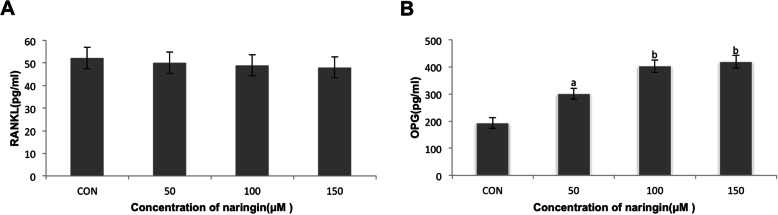
Naringin stimulated OPG secretion by periprosthetic membrane-derived fibroblasts. The fibroblasts were treated with naringin at various concentrations (50 μM, 100 μM, 150 μM) or in medium alone (control group) for 24 h. The levels of **a** RANKL and **b** OPG in the supernatant were determined by ELISA. Naringin did not increase RANKL secretion but enhanced OPG secretion in a dose-dependent manner (ap < 0.01, bp < 0.001)

The fibroblasts were cultured with naringin (50 μM, 100 μM, 150 μM) or only in the medium (control group) for 24 h. As shown in Fig. 3a and b, RANKL secretion was unaffected. OPG protein was enhanced in a dose-dependent manner (ap < 0.01, bp < 0.001).

### DKK1 inhibited the expression of OPG induced by naringin

**Fig. 4:**
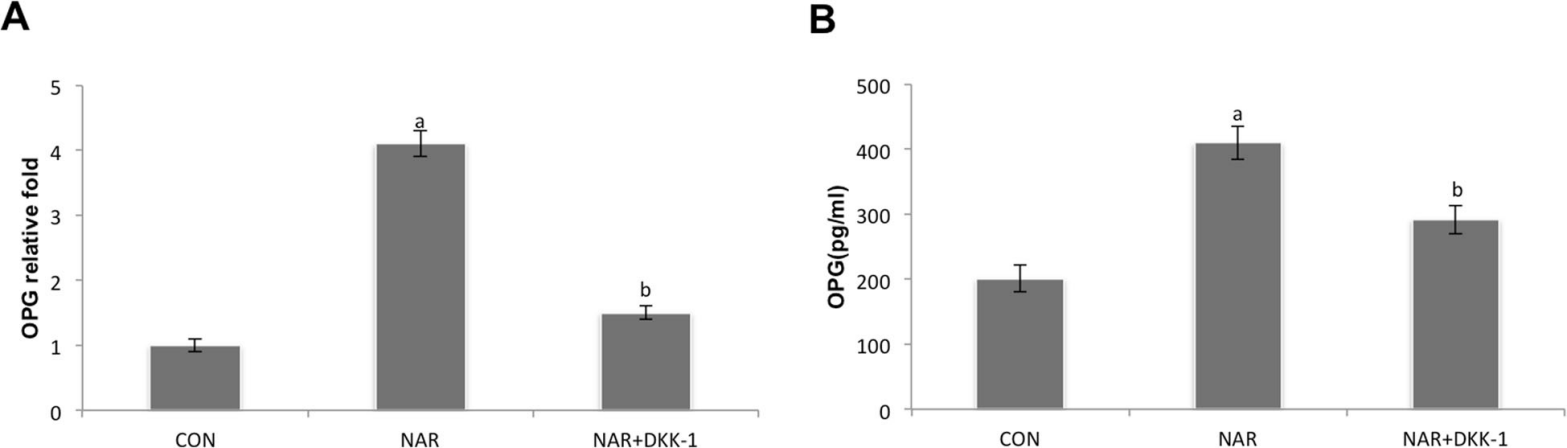
DKK1 inhibited OPG expression by periprosthetic membrane-derived fibroblasts treated with naringin. The fibroblasts were treated with 100 μM naringin in the presence or absence of 100 ng/ml DKK-1. **a** OPG mRNA expression was examined by real-time RT-PCR at 6 h, and **b** OPG in the supernatant were determined by ELISA at 24 h. The canonical Wnt pathway inhibitor DKK1 treatment could markedly attenuate the effects of naringin on OPG (ap < 0.001 versus control group; bp < 0.001 versus 100 μM naringin group)

The fibroblasts were treated with 100 μM naringin in the presence or absence of 100 ng/ml DKK-1. As shown in Fig. 4a and b, the expression of OPG mRNA was determined by real-time RT-PCR at 6 h, and OPG in the supernatant was examined by ELISA at 24 h. The canonical Wnt pathway inhibitor DKK1 treatment could significantly reduce the effects of naringin on OPG (ap < 0.001 versus control group; bp < 0.001 versus 100 μM naringin group).

### Gene expression of Wnt/β-catenin signal pathway components was analyzed by real-time PCR

**Fig. 5:**
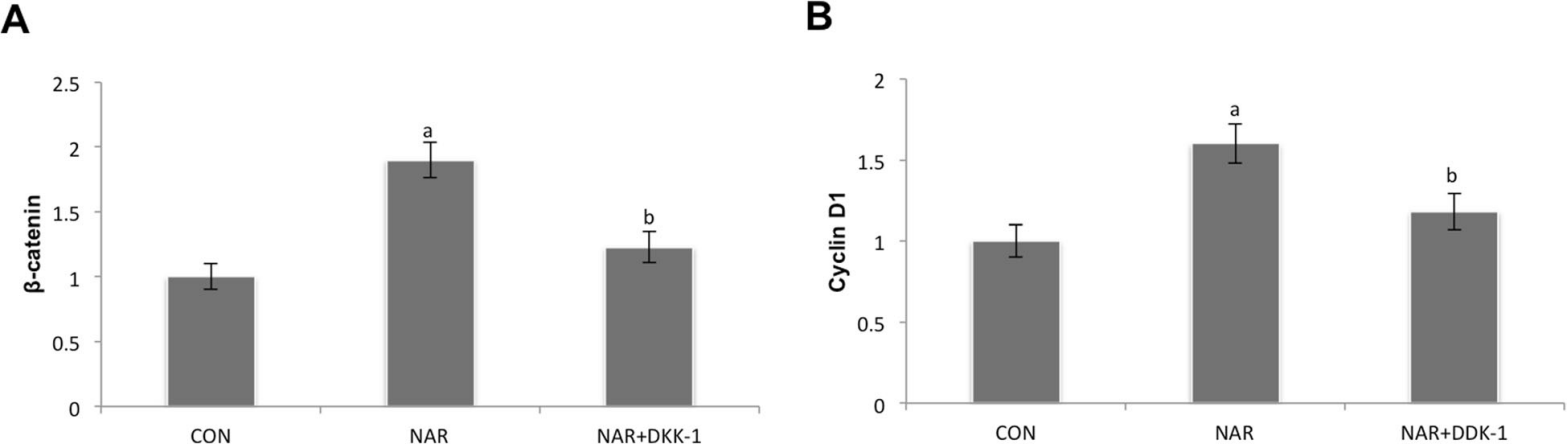
Gene expression of Wnt/b-catenin signal pathway components was analyzed by real-time PCR. The fibroblasts were treated with 100 μM naringin in the presence or absence of 100 ng/ml DKK-1 for 6 h. Transcription of **a** β-catenin and its target gene **b** cyclin D1, which participate in the Wnt signaling pathway, were upregulated by naringin treatment, and this upregulation was markedly attenuated by DDK-1 (ap < 0.001 versus control group; bp < 0.001 versus 100 μM naringin group)

The fibroblasts were treated with 100 μM naringin in the presence or absence of 100 ng/ml DKK-1 for 6 h. As shown in Fig. 5a and b, β-catenin and its target gene cyclin D1 involved in the Wnt signaling pathway were upregulated by naringin treatment, and this upregulation was markedly attenuated by DDK-1 (ap < 0.001 versus control group; bp < 0.001 versus 100 μM naringin group).

## Discussion

Homeostasis of bone metabolism is maintained through a balance between bone resorption and bone formation. When osteoblast bone formation is less than osteoclast bone resorption, aseptic loosening occurs. The ratio of OPG to RANKL controls the process of osteogenesis and osteoclastogenesis [29]. RANKL, produced by BMSCs and osteoblasts, induces osteoclast differentiation and activation by binding to the RANK receptor on osteoclast precursors. Osteoblasts also produce OPG, a soluble “decoy receptor” that blocks the interaction between RANKL and RANK and affects bone metabolism. Therefore, the balance of RANKL and OPG plays an important role in bone metabolic homeostasis. Targeting the RANKL/RANK/OPG system should have an important effect on the differentiation and function of osteoclast. Previous studies have shown that the RANKL expression is elevated in fibroblasts in the periprosthetic membrane around failed prostheses [2]. Therefore, we detected the expression levels of OPG and RANKL in fibroblasts after naringin treatment. Our study demonstrated that naringin obviously enhanced the mRNA and protein levels of OPG. By contrast, naringin had no significant effects on the RANKL mRNA level and RANKL secretion. This suggested that naringin might inhibit osteoclast differentiation by inducing OPG/RANKL ratio, which is consistent with the idea that naringin increased OPG expression in osteoblasts and human amniotic fluid-derived stem cells [20, 24].

Wnt/β-catenin signaling plays a crucial role in bone metabolism. The activation of Wnt/β-catenin signaling pathway has been extensively studied in bone biology and has been proved to promote bone formation. Research showed this pathway played a role in the development of osteolytic disease [30]. The importance of regulation of the Wnt/β-catenin signaling pathway has also been noted in the field of aseptic loosening research [31–33]. β-catenin is an important part of Wnt/β-catenin signaling, which modulates transcription of Wnt target genes, including OPG [34]. It was shown that mice expressing β-catenin in osteoblasts exhibited a high bone mass phenotype by inhibiting osteoclast differentiation and bone resorption [34]. CyclinD1 is considered to be a target of the typical Wnt pathway [35]. In this study, we showed that naringin enhanced the β-catenin and cyclin D1 expression levels, suggesting that naringin may increase OPG by partially upregulating Wnt/β-catenin pathway in fibroblasts. To verify this result, we cultured fibroblasts with naringin and Dkk-1, which specifically inhibited the Wnt/β-catenin pathway and found that DKK-1 had obvious inhibitory effect on OPG, β-catenin, and cyclin D1 expression induced by naringin, which further confirmed OPG expression was enhanced through Wnt/β-catenin pathway.

The agents that increase the activity of the Wnt/beta catenin pathway may serve as promising agents for the treatment of osteolysis. Naringin is a natural compound in citrus fruits, which has been proved to promote bone development and maintenance. The effect of naringin on Wnt/β-catenin signaling has been investigated because Wnt/β-catenin signaling is related to osteoblastogenesis. It was found that naringin stimulated Wnt/β-Catenin signaling in osteoblast-like UMR-106 cells [36], naringin protected human adipose-derived mesenchymal stem cells from oxidative stress-induced inhibition of osteogenic differentiation, which may be associated with Wnt signaling pathway [21], and naringin could prevent the progression of disuse osteoporosis in rats by activating Wnt/β-catenin signaling pathways [37]. Therefore, the effect of naringin on OPG might be mediated through activation of Wnt/β-catenin pathway. A recent study showed that naringin significantly increased the OPG expression in human amniotic fluid-derived stem cells via Wnt/β-catenin signaling pathways [24], which was consistent with our results.

These results indicated that naringin activates Wnt/β-catenin signaling molecules, thereby enhancing the OPG expression in fibroblasts. However, the details of the mechanism of OPG expression induced by naringin are not fully understood. Recent studies have suggested that naringin activity may be mediated not only by the Wnt/β-catenin signaling pathway but also by the NF-κB, ERK, and other signaling pathways [38–40]. Because these pathways may exhibit intricate cross-talk, it is possible that the addition of DKK-1, by inhibiting only the Wnt/β-catenin signaling pathway, did not completely block OPG expression in the present study. In particular, although our findings indicated that naringin influenced OPG expression in large part through Wnt/β-catenin signal transduction in fibroblasts, the general inability of DKK-1 to completely abrogate OPG expression of naringin treatment to the level observed in the control (untreated) suggested that this effect might not be specific to the Wnt/β-catenin signaling pathway. Actually, fibroblasts might have other potential mechanisms responding to naringin. Further studies are therefore necessary to elucidate the precise mechanism of OPG expression induced by naringin.

## Conclusion

In summary, this study showed that naringin might regulate OPG expression through the Wnt/β-catenin signaling pathway in fibroblasts from periprosthetic membrane. Our findings indicated that naringin could be used as a potential therapeutic drug for the treatment of periprosthetic osteolysis and aseptic loosening.

## Data Availability

All data generated or analyzed during this study are included in this published article.

## Abbreviations

RANKL: Activator of nuclear factor kappa B ligand
OPG: Osteoprotegerin
α-MEM: α-Minimal essential medium
CCK-8: Cell Counting Kit-8

## References

[1] Sakai H, Jingushi S, Shuto T, Urabe K, Ikenoue T, Okazaki K (2002). Fibroblasts from the inner granulation tissue of the pseudocapsule in hips at revision arthroplasty induce osteoclast differentiation, as do stromal cells. Ann Rheum Dis..

[2] Crotti TN, Smith MD, Findlay DM, Zreiqat H, Ahern MJ, Weedon H (2004). Factors regulating osteoclast formation in human tissues adjacent to peri-implant bone loss: expression of receptor activator NFkappaB, RANK ligand and osteoprotegerin. Biomaterials..

[3] Mandelin J, Liljeström M, Li TF, Ainola M, Hukkanen M, Salo J (2005). Pseudosynovial fluid from loosened total hip prosthesis induces osteoclast formation. J Biomed Mater Res B Appl Biomater..

[4] Mandelin J, Li TF, Liljeström M, Kroon ME, Hanemaaijer R, Santavirta S (2003). Imbalance of RANKL/RANK/OPG system in interface tissue in loosening of total hip replacement. J Bone Joint Surg Br..

[5] Mandelin J, Li TF, Hukkanen M, Liljeström, Salo J, Santavirta S (2005). Interface tissue fibroblasts from loose total hip replacement prosthesis produce receptor activator of nuclear factor-kappaB ligand, osteoprotegerin, and cathepsin K. J Rheumatol..

[6] Koreny T, Tunyogi-Csapó M, Gál I, Vermes C, Jacobs JJ, Glant TT (2006). The role of fibroblasts and fibroblast-derived factors in periprosthetic osteolysis. Arthritis Rheum..

[7] Ramage SC, Urban NH, Jiranek WA, Maiti A, Beckman MJ (2007). Expression of RANKL in osteolytic membranes: association with fibroblastic cell markers. J Bone Joint Surg Am..

[8] Sabokbar A, Itonaga I, Sun SG, Kudo O, Athanasou NA (2005). Arthroplasty membrane-derived fibroblasts directly induce osteoclast formation and osteolysis in aseptic loosening. J Orthop Res..

[9] Childs LM, Paschalis EP, Xing L, Dougall WC, Anderson D, Boskey AL (2002). In vivo RANK signaling blockade using the receptor activator of NF-kappaB:Fc effectively prevents and ameliorates wear debris-induced osteolysis via osteoclast depletion without inhibiting osteogenesis. J Bone Miner Res..

[10] Ulrich-Vinther M, Carmody EE, Goater JJ, S balle K, O’Keefe RJ, Schwarz EM (2002). Recombinant adeno-associated virus-mediated osteoprotegerin gene therapy inhibits wear debris-induced osteolysis. J Bone Joint Surg Am.

[11] Goater JJ, O'Keefe RJ, Rosier RN, Puzas JE, Schwarz EM (2002). Efficacy of ex vivo OPG gene therapy in preventing wear debris induced osteolysis. J Orthop Res..

[12] Han X, Gong S, Li N, Wang X, Liu P, Xu Y (2019). A novel small molecule which increases osteoprotegerin expression and protects against ovariectomy-related bone loss in eats. Front Pharmacol..

[13] Chen B, Li XD, Liu DX, Wang H, Xie P, Liu ZY (2012). Canonical Wnt signaling is required for Panax notoginseng saponin-mediated attenuation of the RANKL/OPG ratio in bone marrow stromal cells during osteogenic differentiation. Phytomedicine..

[14] Cheng X, Wei B, Sun L, Hu X, Liang J, Chen Y (2016). Astragaloside I stimulates osteoblast differentiation through the Wnt/β-catenin signaling pathway. Phytother Res..

[15] Zhang L, Liu W, Zhao J, Ma X, Shen L, Zhang Y (1860). Mechanical stress regulates osteogenic differentiation and RANKL/OPG ratio in periodontal ligament stem cells by the Wnt/β-catenin pathway. Biochim Biophys Acta..

[16] Qian Y, Zhang XL, Jiang Y, Zeng BF, Wang Q, Chen Y (2015). Alendronate stimulates osteoprotegerin expression in fibroblasts from periprosthetic membrane. Hip Int..

[17] Zhang P, Dai KR, Yan SG, Yan WQ, Zhang C, Chen DQ (2009). Effects of naringin on the proliferation and osteogenic differentiation of human bone mesenchymal stem cell. Eur J Pharmacol..

[18] Li N, Jiang Y, Wooley PH, Xu Z, Yang SY (2013). Naringin promotes osteoblast differentiation and effectively reverses ovariectomy-associated osteoporosis. J Orthop Sci..

[19] Ang ES, Yang X, Chen H, Liu Q, Zheng MH, Xu J (2011). Naringin abrogates osteoclastogenesis and bone resorption via the inhibition of RANKL-induced NF-κB and ERK activation. FEBS Lett..

[20] Xu T, Wang L, Tao Y, Ji Y, Deng F, Wu XH (2016). The function of naringin in inducing secretion of osteoprotegerin and inhibiting formation of osteoclasts. Evid Based Complement Alternat Med..

[21] Wang L, Zhang YG, Wang XM, Ma LF, Zhang YM (2015). Naringin protects human adipose-derived mesenchymal stem cells against hydrogen peroxide-induced inhibition of osteogenic differentiation. Chem Biol Interact..

[22] Fan J, Li J, Fan Q (2015). Naringin promotes differentiation of bone marrow stem cells into osteoblasts by upregulating the expression levels of microRNA-20a and downregulating the expression levels of PPARγ. Mol Med Rep..

[23] Li N, Xu Z, Wooley PH, Zhang J, Yang SY (2013). Therapeutic potentials of naringin on polymethylmethacrylate induced osteoclastogenesis and osteolysis, in vitro and in vivo assessments. Drug Des Devel Ther..

[24] Liu M, Li Y, Yang ST (2017). Effects of naringin on the proliferation and osteogenic differentiation of human amniotic fluid-derived stem cells. J Tissue Eng Regen Med..

[25] Yu X, Zhao X, Wu T, Zhou Z, Gao Y, Wang X (2013). Inhibiting wear particles-induced osteolysis with naringin. Int Orthop..

[26] Qian Y, Zhang XL, Zeng BF, Jiang Y, Shen H, Wang Q (2013). Substance P enhanced titanium particles-induced RANKL expression in fibroblasts from periprosthetic membrane. Connect Tissue Res..

[27] Qian Y, Zeng BF, Zhang XL, Jiang Y (2008). Substance P stimulates production of interleukin 1beta and tumor necrosis factor alpha in fibroblasts from hip periprosthetic membrane. J Arthroplasty..

[28] Qian Y, Zeng BF, Zhang XL, Jiang Y (2007). Substance P augments PGE2 and IL-6 production in titanium particles-stimulated fibroblasts from hip periprosthetic membrane. J Biomed Mater Res A..

[29] Kong YY, Yoshida H, Sarosi I, Tan HL, Timms E, Capparelli C (1999). OPGL is a key regulator of osteoclastogenesis, lymphocyte development and lymph-node organogenesis. Nature..

[30] Gunn WG, Krause U, Lee N, Gregory CA (2011). Pharmaceutical inhibition of glycogen synthetase kinase-3β reduces multiple myeloma-induced bone disease in a novel murine plasmacytoma xenograft model. Blood..

[31] Geng D, Wu J, Shao H, Zhu S, Wang Y, Zhang W (2015). Pharmaceutical inhibition of glycogen synthetase kinase 3 beta suppresses wear debris-induced osteolysis. Biomaterials..

[32] Qu R, Chen X, Yuan Y, Wang W, Qiu C, Liu L (2019). Ghrelin fights against titanium particle-induced inflammatory osteolysis through activation of β-catenin signaling pathway. Inflammation..

[33] Gu Y, Wang Z, Shi J, Wang L, Hou Z, Guo X (2017). Titanium particle-induced osteogenic inhibition and bone destruction are mediated by the GSK-3β/β-catenin signal pathway. Cell Death Dis..

[34] Glass DA, Bialek P, Ahn JD, Starbuck M, Patel MS, Clevers H (2005). Canonical Wnt signaling in differentiated osteoblasts controls osteoclast differentiation. Dev Cell..

[35] Sansom OJ, Reed KR, van de Wetering M, Muncan V, Winton DJ, Clevers H (2005). Cyclin D1 is not an immediate target of beta-catenin following Apc loss in the intestine. J Biol Chem..

[36] Wang D, Ma W, Wang F, Dong J, Wang D, Sun B (2015). Stimulation of Wnt/β-catenin signaling to improve bone development by naringin via interacting with AMPK and Akt. Cell Physiol Biochem..

[37] Lv J, Sun X, Ma J, Ma X, Xing G, Wang Y (2015). Involvement of periostin-sclerostin-Wnt/β-catenin signaling pathway in the prevention of neurectomy-induced bone loss by naringin. Biochem Biophys Res Commun..

[38] Zhao Y, Li Z, Wang W, Zhang H, Chen J, Su P (2016). Naringin protects against cartilage destruction in osteoarthritis through repression of NF-κB signaling pathway. Inflammation..

[39] Wang H, Li C, Li J, Zhu Y, Jia Y, Zhang Y (2017). Naringin enhances osteogenic differentiation through the activation of ERK signaling in human bone marrow mesenchymal stem cells. Iran J Basic Med Sci..

[40] Wei K, Xie Y, Chen T, Fu B, Cui S, Wang Y (2017). ERK1/2 signaling mediated naringin-induced osteogenic differentiation of immortalized human periodontal ligament stem cells. Biochem Biophys Res Commun..

